# A comparison of methods for estimating the temporal change in a continuous variable: Example of HbA1c in patients with diabetes

**DOI:** 10.1002/pds.4273

**Published:** 2017-08-15

**Authors:** Therese Sheppard, Robyn Tamblyn, Michal Abrahamowicz, Mark Lunt, Matthew Sperrin, William G. Dixon

**Affiliations:** ^1^ Division of Musculoskeletal and Dermatological Sciences, Arthritis Research UK Centre for Epidemiology, School of Biological Sciences, Faculty of Biology, Medicine and Health The University of Manchester, Manchester Academic Health Science Centre Manchester UK; ^2^ Department of Epidemiology, Biostatistics and Occupational Health McGill University Quebec Canada; ^3^ Department of Medicine McGill University Quebec Canada; ^4^ Clinical and Health Informatics Research Group McGill University Quebec Canada; ^5^ Centre for Health Informatics, Division of Informatics, Imaging and Data Sciences, School of Health Sciences, Faculty of Biology, Medicine and Health The University of Manchester, Manchester Academic Health Science Centre Manchester UK; ^6^ Health e‐Research Centre, Farr Institute The University of Manchester, Manchester Academic Health Science Centre Manchester UK

**Keywords:** continuous variable, functional principal component analysis, linear interpolation, mean prediction error, pharmacoepidemiology, predictive accuracy, sparse longitudinal data

## Abstract

**Purpose:**

To compare the more complex technique, functional principal component analysis (FPCA), to simpler methods of estimating values of sparse and irregularly spaced continuous variables at given time points in longitudinal data using a diabetic patient cohort from UK primary care.

**Methods:**

The setting for this study is the Clinical Practice Research Datalink (CPRD), a UK general practice research database. For 16,034 diabetic patients identified in CPRD, with at least 2 measures in a 30‐month period, HbA1c was estimated after temporarily omitting (i) the final and (ii) middle known values using linear interpolation, simple linear regression, arithmetic mean, random effects, and FPCA. Performance of each method was assessed using mean prediction error. The influence on predictive accuracy of (1) more homogeneous populations and (2) number and range of known HbA1c values was explored.

**Results:**

When estimating the last observation, the predictive accuracy of FPCA was highest with over half of predicted values within 0.4 units, equivalent to laboratory measurement error. Predictive accuracy improved when estimating the middle observation with almost 60% predicted values within 0.4 units for FPCA. These results were marginally better than that achieved by simpler approaches, such as last‐occurrence‐carried‐forward linear interpolation. This pattern persisted with more homogeneous populations as well as when variability in HbA1c measures coupled with frequency of data points were considered.

**Conclusions:**

When estimating change from baseline to prespecified time points in electronic medical records data, a marginal benefit to using the more complex modelling approach of FPCA exists over more traditional methods.

## 1 INTRODUCTION

Opportunities for research using routinely collected data will increase significantly over coming years with expansion of electronic health records (EHR), and investment in e‐infrastructure for research, distributed data networks, and patient‐centred research.^1^, ^2^, ^3^ Analysis of data collected primarily for healthcare delivery rather than research generates methodological challenges. Progress is happening in many areas, for example, studying the same question across different geographical settings^4^ with different healthcare systems and adjusting for confounders defined and measured differently in different settings.^3^ Less attention has been paid to the challenge of dealing with data collected at irregular time intervals.

One significant challenge in real‐life studies of drug use is defining an effectiveness outcome comparable between individual patients, given the varied patterns of assessment timing. In clinical trials, patients are invited for assessment at prespecified intervals. In reality, patients visit their doctor at any time. It thus becomes impossible to measure change in glycosylated haemoglobin (HbA1c) at any given week unless the patient has visited their doctor then. Yet if we want to use observational research to assess effectiveness of oral hypoglycaemics (OHG), we should compare changes in HbA1c over an agreed time interval. Options for estimating values of continuous variables at given time points exist such as selecting the closest temporal measurement as a surrogate,^5^ linear interpolation or ‘joining‐the‐dots’ assuming linear change between each sequential measurement,^6^ averaging measures over yearly intervals^7^ or estimating simple linear regression (SLR) lines using 2 or more measurements. More complex techniques are also available, for instance, random effects (RE) modelling, which allows for population and individual variations. Certain methods consider multiple imputation for longitudinal data^8^ but require data to be measured at regular time points, thus not applicable in this context. A nonparametric statistical technique known as functional principal component analysis (FPCA) exists to model longitudinal data,^9^ although not widely used in epidemiology. This approach views longitudinal data records as functions, where each curve is a single observation, but the belief is that data are sampled from a process, which is continuous over time. Statistical emphasis is shifted onto observed data functions and no longer on individual observations.^10^ The technique's aim is to develop a continuous‐in‐time estimation (or ‘trajectory’) of a continuous variable, based on the individual's own data points as well as patterns of change within the whole population.

It is unclear whether more complex techniques perform better when dealing with sparse and irregularly spaced data. Hypothetically, simpler models may work well when variability of measurements is limited, but more complex models may work better in certain situations. The study's aim was to compare FPCA methodology to other simpler methods in a cohort of diabetic patients from primary care. Specific objectives included (1) removing known HbA1c observations and calculating prediction error by comparing estimated to known values at specific time points in the whole cohort as well as in treatment, gender, and age strata and (2) exploring whether methods perform differently in certain circumstances, such as when there are changes in number of medications, length of time between consecutive measurements, and data sparseness levels within stable and unstable disease groupings.

**KEY POINTS**

- Measuring outcomes in observational studies at prespecified time intervals is difficult when data are sparse and irregularly spaced.
- Traditional methods of estimation include techniques, such as last‐observation‐carried‐forward linear interpolation, which allow crude estimation of change in continuous variables at specified time points (eg, change at 1 year).
- More complex techniques exist, such as FPCA, to model sparse longitudinal data.
- In patients with diabetes, this study demonstrates that in the setting of sparse and irregularly spaced data, using the more complex method, FPCA, has a marginal benefit.

## 2 METHODS

### 2.1 Study population

The setting was the Clinical Practice Research Datalink (CPRD), a UK database of anonymised primary care EHRs covering an active population of over 8 million people.^11^, ^12^

**Figure 1:**
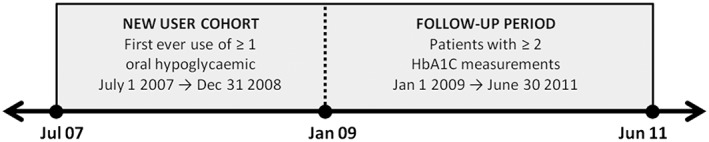
Establishment of cohort of new users of oral hypoglycaemics during July 2007 – January 2009, with index date of 1 January 2009 and 30‐month follow‐up period from January 2009 – June 2011

Adult patients with type 2 diabetes defined by Read codes (code list available from the authors) or who were prescribed OHG medication between 1987 and July 2011 were identified from CPRD. Practices were excluded if their last collection date preceded the study end date or the practice did not meet minimum data quality standards, as assessed by CPRD, throughout the study period. We restricted analysis to new users of OHG medication in the period 1 July 2007 to 31 December 2008 (defined as first ever use of an OHG) in order to generate a more homogeneous cohort. Patients were then required to have at least 2 HbA1c measurements in the study period 1 January 2009 to 30 June 2011 (Figure 1). The UK Quality and Outcomes Framework incentivises general practitioners to measure HbA1c at least once every 15 months for diabetic patients,^13^ meaning most patients had 2 or more measurements in our 2 1/2‐year study window. Patients who died or transferred out of practice, (thus not eligible for the full 30‐month follow‐up) were excluded. The study was approved by the Independent Scientific Advisory Committee of CPRD (ref 11_154A).

### 2.2 Statistical analysis

**Figure 2:**
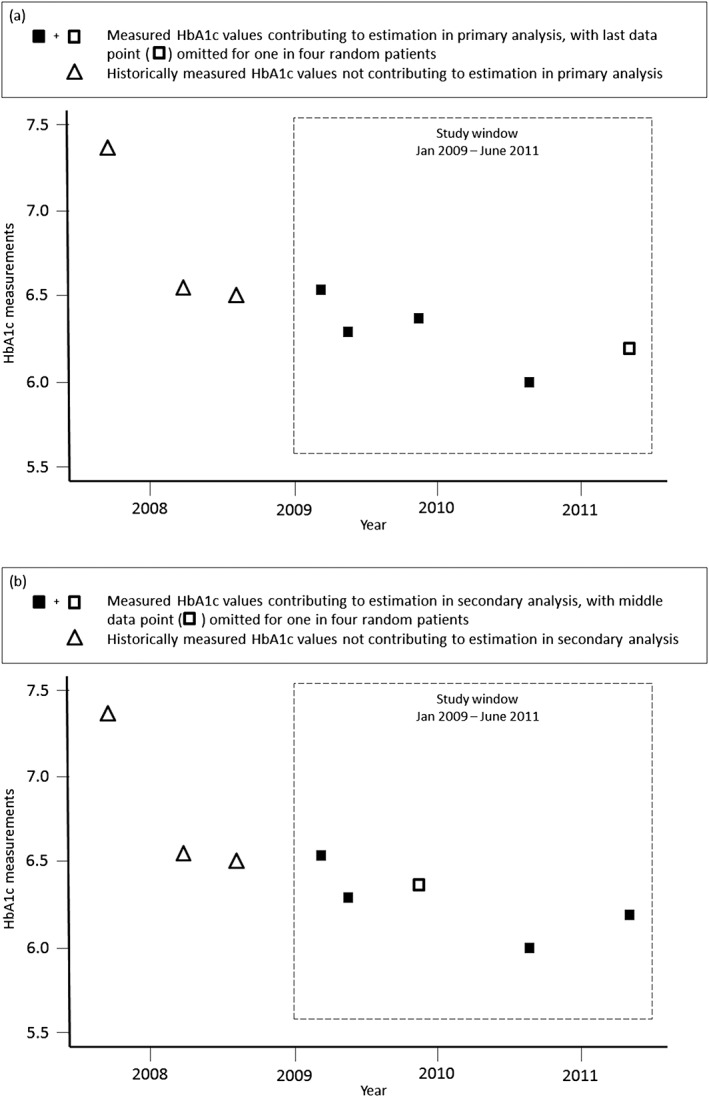
Schematic of a single patient's HbA1c measurements since first ever using oral hypoglycaemic medication, where triangles and squares represent true measured values

A primary analysis used all available HbA1c observations within the study period after temporarily excluding the final data point for 1‐in‐4 randomly selected patients (Figure 2a). The temporarily excluded data point was later reinserted and used to calculate (1) prediction error defined as the absolute difference between the actual HbA1c measurement and its estimated value (*d*) and (2) squared prediction error. This was developed to allow estimation of prediction error at times when outcomes for some patients may not have been measured and could not contribute to the estimation. The procedure of removing a random 25% of final data points was done 6 times to reflect the variability that would occur depending on which data points were sampled. Results across the 6 data sets were pooled with mean and SD values for each measure of predictive accuracy calculated. Coefficients of variation (CV), defined as the ratio of SD to the mean, were also generated as a measure of precision between the 6 replicated data sets.

Linear interpolation methods were used in the primary analysis when estimating the final observation for each individual, as described by Genolini,^14^ and summarised in Table 1.

**Table 1:** Definition of linear interpolation methods to impute missing observations

*For missing values at the end*	
Last occurrence carried forward (LOCF)	Missing value imputed from the previous nonmissing value (Figure 3a)
Global	Missing value imputed by prolonging a line joining the first and last nonmissing values (Figure 3b)
Local	Missing value imputed locally by prolonging a line joining the penultimate and last nonmissing values (Figure 3c)
Bisector	Missing value imputed from an intermediate line, the bisector, between the global and local lines (Figure 3d)
*For missing values in the middle*	
Last occurrence carried forward (LOCF)	Missing value imputed from the previous nonmissing value (Figure 3a)
Next occurrence carried backward (NOCB)	Missing value imputed from the next nonmissing value (Figure 3h)
Global	Missing value imputed by drawing a line between first and last nonmissing values (Figure 3i)
Local	Missing value imputed by drawing a line between the nonmissing values immediately surrounding the missing one (Figure 3j)

**Figure 3:**
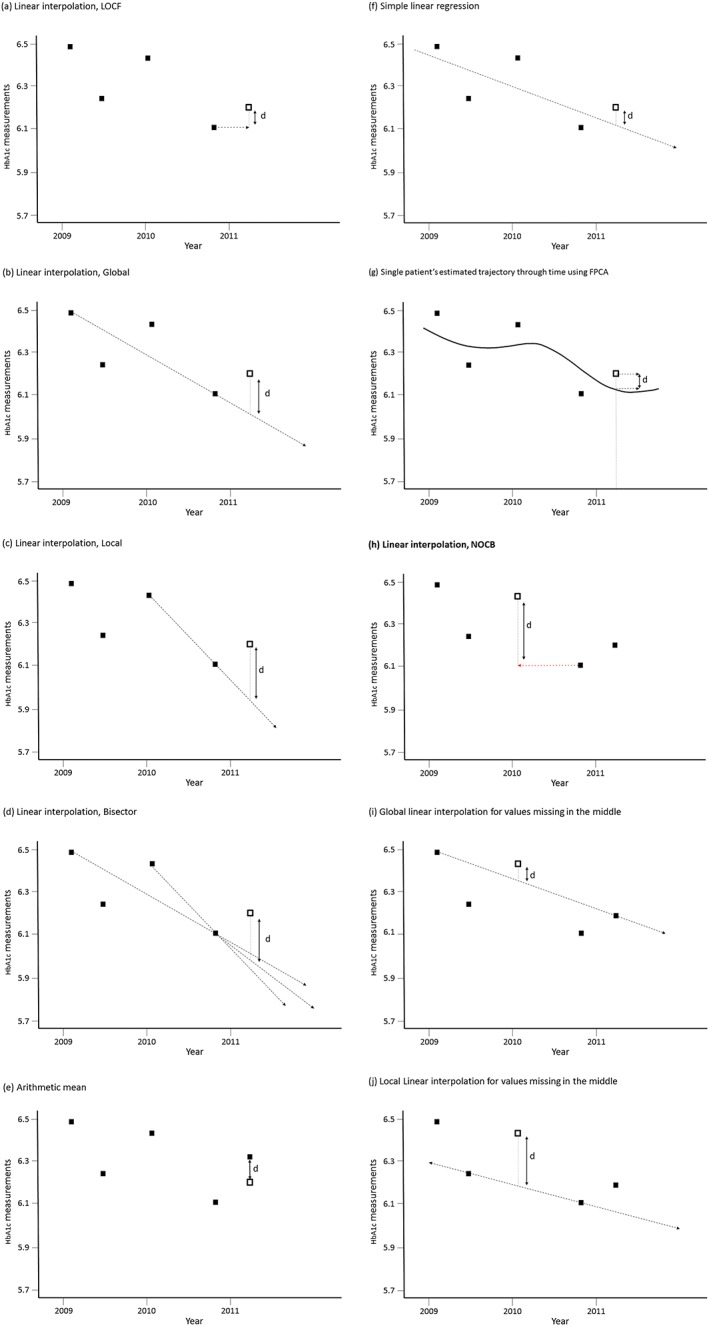
Various definitions of estimation methods. (a) LOCF indicates last occurrence carried forward linear interpolation. (b) Global linear interpolation. (c) Local linear interpolation. (d) Bisector linear interpolation. (e) Arithmetic mean method. (f) Simple linear regression. (g) FPCA indicates functional principal component analysis. When imputing values missing in the middle: (h) NOCB, next occurrence carried backward; (i) global linear interpolation, (j) local linear interpolation [Colour figure can be viewed at wileyonlinelibrary.com]

As an alternative to linear interpolation methods, the arithmetic mean (AM) method was used, which involved simply calculating the average of the nonmissing HbA1c measures for that individual (Figure 3e).

Another computationally simple method considered was fitting a regression line in an SLR using each individual's set of nonmissing HbA1c measures as the outcome variable and time as the predictor variable where each individual's missing last value was subsequently estimated from this fitted line (Figure 3f).

One other estimation approach of temporarily excluded values was the use of RE modelling using random intercepts and constant slope. Individuals once again were not assumed to be measured at the same number of time points, but rather at different time points. This model estimates the individual's values across time on the basis of whatever data that individual has, enhanced by the time trend that is estimated for the sample as a whole but with the added bonus of taking into account the effects of covariates, age and gender, in the model.^15^

A final approach was to use FPCA methodology in developing patient‐specific estimated trajectories, using all data from the whole population, which would then allow estimation of a continuous variable, such as HbA1c, for each individual, not only at the last data point but also at any time point of interest throughout the study period (Figure 3g). It is the only method tested that allows for the possibility that HbA1c changes nonlinearly with time, or that patterns of change differ between individuals.

All interpolation methods, including AM and SLR, estimated temporarily excluded values using just that individual's set of data, whereas with the model‐based approaches of RE and FPCA, it was necessary to use data available on all subjects in the study cohort when making estimations at specific time points for particular individuals.

A secondary analysis sought to examine whether prediction errors improved for any estimation method by removing the middle data point for the same 6 sets of 1‐in‐4 randomly selected patients (Figure 2b). This analysis allows us to use the additional linear interpolation method, next occurrence carried backward (NOCB) (Table 1 and Figure 3h). Global and local interpolation methods are slightly modified in this analysis as shown in Table 1 and Figures 3i and 3j.

Predictive accuracy of each method was assessed using mean absolute prediction error (MAPE), defined as the average absolute prediction error across the quarter of patients where either their last data points in the primary analysis or their middle data points in the secondary analysis were omitted from the estimation. The distribution of squared prediction errors was examined and mean squared absolute prediction error (MSAPE) was used in method comparisons.

To appreciate the importance of the difference between estimated and true HbA1c, we calculated the proportion of individual absolute prediction errors that were (i) below measurement error and (ii) below a clinically meaningful difference. HbA1c measurement error is considered to be around 0.4% assuming an average HbA1c value of 8%.^16^ We defined the clinically meaningful difference as the change in HbA1c associated with a 10% increased risk of any endpoint related to diabetes, which equates to a change in HbA1c of 0.5%.^17^

The lowest values for MAPE and MSAPE and the highest proportion of absolute prediction errors (i) within a clinically meaningful difference and (ii) within measurement error, indicated the optimal estimation method.

### 2.3 Factors influencing prediction error

We anticipated that prediction error would be affected by many factors, such as medication, gender, age, switching medication, distance in time between missing HbA1c measure and its nearest neighbour, and density of HbA1c measures coupled with disease stability. Therefore, after first applying all estimation methods to the whole new user cohort, we performed separate analyses based on the following subgroups:

Stratum A metformin‐only new users
Stratum B female new users
Stratum C female metformin‐only new users
Stratum D female metformin‐only new users aged (i) ≤58, (ii) 59–70, or (iii) ≥71 years
Stratum E new users with medication changes (i) single and (ii) ≥2
Stratum F new users whose distance in time between missing HbA1c measure and its closest neighbour (i) >50th percentile (ii) >75th percentile and (iii) >90th percentile
Stratum G new users with 2–3, 4–5, 6–8, or >8 HbA1c measures in the study window and for whom their range of recorded values is <2 or ≥2 HbA1c units (representing stable and unstable disease, respectively)

These sensitivity analyses were done only for a single replicated data set. With each stratum, we created a homogeneous population and as such we expected modelling methods to perform better than other approaches that do not take into account population behaviour. The exception to this is likely to be stratum G, where we restricted the population to patients with highly variable HbA1c and as such the expectation was that all approaches may underperform.

The analysis was conducted using Stata V.12.1 (http://www.stata.com) and R V.3.1.3 (http://www.R-project.org).

## 3 RESULTS

Between 1987 and July 2011, 500,643 adult patients were identified to have either a Read code for diabetes or any prescribed OHG medication. After excluding approximately 480,000 preexisting users, there were 20,570 patients found to be new users of this medication type from 1 July 2007 to 31 December 2008 having also limited to practices that were ‘up‐to‐standard’ throughout follow‐up and excluding patients who died or transferred out of practice prior to 30 June 2011. Of these, 16,034 patients had 2 or more measures between 1 January 2009 and 30 June 2011. These patients comprise the whole new user cohort.

Mean age at study start was 61 years and 43% of patients were female. The number of HbA1c measurements per individual ranged from 2 to 17 with a median of 4 (interquartile range, 3–6). The median period between measurements was 163 days (interquartile range, 104–221).

Table 2 shows results of the primary analysis after applying all estimation approaches to all 6 data sets, in which individuals were randomly selected to have their last HbA1c observation temporarily dropped from the whole new user cohort. CVs show that the sampled data sets have low variability because SDs are small compared to their corresponding means. This suggests that because of the precision of just 6 replicated data sets, any differences in performance are real and not merely due to random variation.

**Table 2:** Prediction error (absolute difference between predicted and actual values of last observation), described as MAPE and root MSAPE of 4009 subjects and proportion of these subjects with predictions within clinical acceptability and measurement error. Reporting pooled results of 6 randomly sampled data sets from whole new user cohort using mean, SD, and CV

	LOCF	Global	Local	Bisector	AM	SLR	RE‐no covs	RE‐withcovs***	FPCA
*MAPE*									
Mean	0.60	1.63	1.63	1.59	0.66	0.96	0.65	0.65	0.59
SD	0.009	0.010	0.033	0.023	0.009	0.016	0.005	0.008	0.008
CV	1.5%	0.6%	2%	1.45%	1.36%	1.67%	0.77	1.23%	1.36%
*Proportion of absolute prediction errors <0.5* *									
Mean	60%	37%	37%	34%	55%	49%	57%	57%	61%
SD	0.516	0.816	0.984	0.816	0.753	0.753	0.837	0.516	1.033
CV	0.86%	2.21%	2.66%	2.4%	1.37%	1.54%	1.47%	0.91%	1.69%
*Proportion of absolute prediction errors <0.4* **									
Mean	52%	32%	31%	29%	48%	42%	49%	49%	54%
SD	0.753	0.753	0.753	0.753	0.894	0.516	0.548	0.408	0.548
CV	1.45%	2.35%	2.43%	2.6%	1.86%	1.23%	1.12%	0.83%	1.01%
*Root MSAPE*									
Mean	0.95	2.93	3.40	2.84	0.99	1.85	0.97	0.97	0.91
SD	0.018	0.093	0.445	0.203	0.012	0.164	0.011	0.012	0.010
CV	1.84%	3.15%	13.09%	7.15%	1.25%	8.88%	1.11%	1.26%	1.13%

* Clinically important difference.

** Measurement error.

*** Age and gender.

LOCF indicates last occurrence carried forward; Global, Local and Bisector, linear interpolation methods for value missing at the end; AM, arithmetic mean method; SLR, simple linear regression method; RE‐no covs: random effects modelling method with no covariates included; RE‐with covs: random effects modelling method with covariates included; FPCA, functional principal component analysis method.

The best performance was achieved by FPCA where a mean of 54% of subjects had prediction errors less than measurement error compared to a mean of 29% with bisector linear interpolation, the least performing method. FPCA was only marginally better than last‐occurrence‐carried‐forward (LOCF), RE, and AM approaches (Table 2).

Limiting prediction error assessment to strata A‐F, based on a single data set having removed 1‐in‐4 final data points, produced results that can be seen in Figures S1a – S1d. The overall performance of approaches did not change in pattern from that seen in the whole new user cohort, in that LOCF, AM, RE, and FPCA approaches were optimal followed by SLR, with the remaining linear interpolation methods performing worst. Similar results for stratum G (Figures S2a – S2d) found the pattern of prediction errors within subgroups remained the same, with LOCF, AM, RE, and FPCA generating more accurate predictions. See Table S1 for a summarised version of these results.

Table 3 displays results following removal of the middle data point, where the best performance was achieved once again by FPCA, although closely followed by SLR, AM, and RE, whereas low CVs reflect the fact that these differences in performance are not due to random variation.

**Table 3:** Prediction error (absolute difference between predicted and actual values of middle observation), described as MAPE and root MSAPE of 4009 subjects and proportion of these subjects with predictions within clinical acceptability and measurement error. Reporting pooled results of 6 randomly sampled data sets from whole new user cohort using mean, SD, and CV

	LOCF	NOCB	Global	Local	AM	SLR	RE‐no covs	RE‐with covs***	FPCA
*MAPE*									
Mean	0.62	0.62	0.93	0.67	0.55	0.53	0.56	0.56	0.52
SD	0.010	0.009	0.010	0.008	0.005	0.006	0.005	0.005	0.005
CV	1.61%	1.45%	1.08%	1.19%	0.91%	1.13%	0.89%	0.89%	0.96%
*Proportion of absolute prediction errors <0.5* *									
Mean	58%	58%	51%	61%	63%	65%	63%	63%	66%
SD	0.548	1.095	0.816	0.837	0.516	0.408	0.632	0.632	0.753
CV	0.94%	1.89%	1.6%	1.37%	0.82%	0.63%	1%	1%	1.14%
*Proportion of absolute prediction errors <0.4* **									
Mean	51%	51%	44%	54%	56%	58%	54%	54%	57%
SD	0.408	0.632	0.00	0.548	0.408	0.408	0.516	1.472	0.753
CV	0.8%	1.24%	0%	1.01%	0.73%	0.70%	0.96%	2.73%	1.32%
*Root MSAPE*									
Mean	1.02	1.00	1.53	1.13	0.85	0.83	0.84	0.84	0.80
SD	0.024	0.014	0.026	0.029	0.009	0.017	0.010	0.010	0.010
CV	2.36%	1.39%	1.69%	2.57%	1.08%	2.04%	1.19%	1.19%	1.29%

* Clinically important difference.

** Measurement error.

*** Age and gender.

LOCF indicates last occurrence carried forward; NOCB, next occurrence carried backward; Global and Local, linear interpolation methods for value missing in the middle; AM, arithmetic mean method; SLR, simple linear regression method; .RE‐no covs, random effects modelling method with no covariates included; RE‐with covs, random effects modelling method with covariates included; FPCA, functional principal component analysis method.

In general, a similar pattern of prediction error was found from all approaches in this secondary analysis when limiting prediction error assessment to strata A‐G based on a single data set having removed 1‐in‐4 middle data points (Figures S3a – S3d, Figures S4a – S4d and summarised in Table S2) in that AM, SLR, RE, and FPCA generate the most accurate predictions, with FPCA displaying a marginal benefit.

## 4 DISCUSSION

This study compared methods for estimating values of a continuous variable, HbA1c, at a given time point using known values of this sparse and irregularly spaced data point within UK primary care records of patients with diabetes. Few studies exist, which investigate the effectiveness of these methods, yet researchers apply them without considering their performance.

In Table 2, when estimating the last observation in the primary analysis, LOCF and FPCA proved to be optimal approaches, with FPCA performing marginally better in some assessments, whereas the remaining linear interpolation methods were equally poor. As the populations were made more homogeneous, such as restricting to females or by having single continuous drug use, the more complex modelling involved in RE and FPCA approaches did not outperform the simpler LOCF method, although FPCA achieved slightly better results overall. For example, under FPCA, 59% of female subjects achieved prediction errors below measurement error compared with 54% under LOCF, whereas the least performing method, bisector linear interpolation, only achieved 32%. We expected an optimal performance from FPCA because of its flexibility to deal with longitudinally nonlinear changes in HbA1c, yet the advantage in using this approach was only marginal. We could also have extended SLR to allow for nonlinear effects, but this would have involved making assumptions that were unjustifiable.

When populations are suffering from complexities of sparse (only a few HbA1c measures) and erratic data (large HbA1c range), more flexible modelling methods do not convincingly produce better results. In fact, by looking at levels of prediction error, when we get into sparse and erratic data, we cannot trust any method in that all methods do quite badly. Evidence of this can be seen in the subgroup of new users with 2–3 measures and unstable disease where the proportion of subjects with prediction errors less than the clinical important difference of 0.5 is at best 20% with LOCF.

This study exhibits a couple of important strengths. Approximately 16,000 subjects in the original population provide for a very large study cohort, facilitating the estimation of temporarily excluded HbA1c values using a whole range of techniques, including linear interpolation approaches as well as more complex modelling approaches. Also, knowing what the true measurements are for the ones we are trying to predict allows us to effectively calibrate results.

There are, of course, some limitations. First, we have 1 very particular continuous outcome variable in HbA1c and 1 very particular population in diabetic patients. So our findings may not be generalisable to different populations where there may be different variability through time and different associations with other variables. Second, HbA1c has its own within‐person variability. So it may very well be that for other outcomes, such as weight, which varies more slowly, these estimation approaches may perform differently. Third, it is very likely that HbA1c measurements in this population are missing not at random and unfortunately there is no method of testing available to address this problem. Although we are predicting observed values from observed values, it is possible that missing data would have enabled the more complex modelling methods to work better. Fourth, LOCF, NOCB, and AM all assume the outcome does not change with time, whereas RE and SLR both assume it changes linearly, and FPCA allows for nonlinear changes. When deciding therefore which estimation method is most appropriate, consideration should be given to whether the data violate any underlying assumptions. Fifth, these methods do not account for uncertainty associated with missing values and so using them will produce estimates that are too precise and will lead to potential bias in subsequent analyses. Finally, underrepeated sampling, using the whole cohort only, was justified since the vast majority of CVs were below 3%. However, it is an assumption that as a consequence there would be no change in the order of each method's performance for strata A‐G.

In summary, we have shown that there is a marginal benefit to using the more complex FPCA model when estimating missing HbA1c values in a cohort of patients with diabetes, as results for this model are generally better than all other approaches. However, FPCA is a significantly more complex technique to implement, although worth considering due to the problems attached to using more simple approaches. Caution is needed in extrapolating these findings to other settings as the most appropriate method when estimating values at given time points will likely depend on the variable of interest, the population in which it is measured, where the missing data actually exist, how homogeneous the population is to start with, and the behaviour of patients and clinicians.

### RESEARCH SPONSORS

This study was supported by the Canadian Institutes of Health Research (CIHR) operating grant no. 201009MOP, the Medical Research Council (MRC) Clinician Scientist Fellowship grant no. G0902272, and MRC grant no. MR/K006665/1.

### ETHICS STATEMENT

Study approval was granted by the Independent Scientific Advisory Committee of CPRD (ref 11_154A).

## CONFLICT OF INTEREST

There are no conflicts of interest to disclose.

## Supporting information


**Table S1.** Mean absolute prediction errors (MAPE), proportions of subjects with absolute prediction errors (APE) within clinical acceptability and measurement error, and mean squared absolute prediction errors (MSAPE), for each of eight strata. Reporting on subjects whose last HbA1c value excluded from analysis.
**Figure S1a.** Mean absolute prediction errors for strata (A) new users on metformin only medication, (B) female new users, (C) female metformin‐only new users, (D) female metformin‐only new users aged a) ≤ 58, b) 59–70, c) ≥ 71 years, (E) new users with a) single medication change and b) two or more changes in medication, (F) new users whose distance in time between missing HbA1c measure and its closest neighbour was a) > 50^th^ percentile b) > 75^th^ percentile and c) > 90^th^ percentile, where prediction error is absolute difference between predicted and actual values of last HbA1c observation and n is number of subjects being reported on, whose last value was excluded.
**Figure S1b.** Proportion (%) with absolute prediction errors < 0.5 (clinical important difference) for strata (A) new users on metformin only medication, (B) female new users, (C) female metformin‐only new users, (D) female metformin‐only new users aged a) ≤ 58, b) 59–70, c) ≥ 71 years, (E) new users with a) single medication change and b) two or more changes in medication, (F) new users whose distance in time between missing HbA1c measure and its closest neighbour was a) > 50^th^ percentile b) > 75^th^ percentile and c) > 90^th^ percentile, where prediction error is absolute difference between predicted and actual values of last HbA1c observation and n is number of subjects being reported on, whose last value was excluded.
**Figure S1c.** Proportion (%) with absolute prediction errors < 0.4 (measurement error) for strata (A) new users on metformin only medication, (B) female new users, (C) female metformin‐only new users, (D) female metformin‐only new users aged a) ≤ 58, b) 59–70, c) ≥ 71 years, (E) new users with a) single medication change and b) two or more changes in medication, (F) new users whose distance in time between missing HbA1c measure and its closest neighbour was a) > 50^th^ percentile b) > 75^th^ percentile and c) > 90^th^ percentile, where prediction error is absolute difference between predicted and actual values of last HbA1c observation and n is number of subjects being reported on, whose last value was excluded.
**Figure S1d.** Mean squared absolute prediction errors for strata (A) new users on metformin only medication, (B) female new users, (C) female metformin‐only new users, (D) female metformin‐only new users aged a) ≤ 58, b) 59–70, c) ≥ 71 years, (E) new users with a) single medication change and b) two or more changes in medication, (F) new users whose distance in time between missing HbA1c measure and its closest neighbour was a) > 50^th^ percentile b) > 75^th^ percentile and c) > 90^th^ percentile, where prediction error is absolute difference between predicted and actual values of last HbA1c observation and n is number of subjects being reported on, whose last value was excluded.
**Figure S2a.** Mean absolute prediction errors for subgroups (from stratum G) of new users with 2–3, 4–5, 6–8 or >8 HbA1c measures for both stable (< 2 HbA1c units) and unstable disease (≥ 2 HbA1c units), where prediction error is absolute difference between predicted and actual values of last HbA1c observation and n is number of subjects being reported on, whose last value was excluded.
**Figure S2b.** Proportion (%) with absolute prediction errors < 0.5 (clinical important difference) for subgroups (from stratum G) of new users with 2–3, 4–5, 6–8 or >8 HbA1c measures for both stable (< 2 HbA1c units) and unstable disease (≥ 2 HbA1c units), where prediction error is absolute difference between predicted and actual values of last HbA1c observation and n is number of subjects being reported on, whose last value was excluded.
**Figure S2c.** Proportion (%) with absolute prediction errors < 0.4 (measurement error) for subgroups (from stratum G) of new users with 2–3, 4–5, 6–8 or >8 HbA1c measures for both stable (< 2 HbA1c units) and unstable disease (≥ 2 HbA1c units), where prediction error is absolute difference between predicted and actual values of last HbA1c observation and n is number of subjects being reported on, whose last value was excluded.
**Figure S2d.** Mean squared absolute prediction errors for subgroups (from stratum G) of new users with 2–3, 4–5, 6–8 or >8 HbA1c measures for both stable (< 2 HbA1c units) and unstable disease (≥ 2 HbA1c units), where prediction error is difference between absolute predicted and actual values of last HbA1c observation and n is number of subjects being reported on, whose last value was excluded.
**Table S2.** Mean absolute prediction errors (MAPE), proportions of subjects with absolute prediction errors (APE) within clinical acceptability and measurement error, and mean squared absolute prediction errors (MSAPE), for each of eight strata. Reporting on subjects whose middle HbA1c value excluded from analysis.
**Figure S3a.** Mean absolute prediction errors for strata (A) new users on metformin only medication, (B) female new users, (C) female metformin‐only new users, (D) female metformin‐only new users aged a) ≤ 58, b) 59–70, c) ≥ 71 years, (E) new users with a) single medication change and b) two or more changes in medication, (F) new users whose distance in time between missing HbA1c measure and its closest neighbour was a) >50^th^ percentile b) >75^th^ percentile and c) >90^th^ percentile, where prediction error is absolute difference between predicted and actual values of middle HbA1c observation and n is number of subjects being reported on, whose middle value was excluded.
**Figure S3b.** Proportion (%) with absolute prediction errors < 0.5 (clinical important difference) for strata (A) new users on metformin only medication, (B) female new users, (C) female metformin‐only new users, (D) female metformin‐only new users aged a) ≤ 58, b) 59–70, c) ≥ 71 years, (E) new users with a) single medication change and b) two or more changes in medication, (F) new users whose distance in time between missing HbA1c measure and its closest neighbour was a) > 50^th^ percentile b) > 75^th^ percentile and c) > 90^th^ percentile, where prediction error is absolute difference between predicted and actual values of middle HbA1c observation and n is number of subjects being reported on, whose middle value was excluded.
**Figure S3c.** Proportion (%) with absolute prediction errors < 0.4 (measurement error) for strata (A) new users on metformin only medication, (B) female new users, (C) female metformin‐only new users, (D) female metformin‐only new users aged a) ≤ 58, b) 59–70, c) ≥ 71 years, (E) new users with a) single medication change and b) two or more changes in medication, (F) new users whose distance in time between missing HbA1c measure and its closest neighbour was a) > 50^th^ percentile b) > 75^th^ percentile and c) > 90^th^ percentile, where prediction error is absolute difference between predicted and actual values of middle HbA1c observation and n is number of subjects being reported on, whose middle value was excluded.
**Figure S3d.** Mean squared absolute prediction errors for strata (A) new users on metformin only medication, (B) female new users, (C) female metformin‐only new users, (D) female metformin‐only new users aged a) ≤ 58, b) 59–70, c) ≥ 71 years, (E) new users with a) single medication change and b) two or more changes in medication, (F) new users whose distance in time between missing HbA1c measure and its closest neighbour was a) > 50^th^ percentile b) > 75^th^ percentile and c) > 90^th^ percentile, where prediction error is absolute difference between predicted and actual values of middle HbA1c observation and n is number of subjects being reported on, with middle value excluded.
**Figure S4a.** Mean absolute prediction errors for subgroups (from stratum G) of new users with 2–3, 4–5, 6–8 or >8 HbA1c measures for both stable (< 2 HbA1c units) and unstable disease (≥ 2 HbA1c units), where prediction error is absolute difference between predicted and actual values of middle HbA1c observation and n is number of subjects being reported on, whose middle value was excluded.
**Figure S4b.** Proportion (%) with absolute prediction errors < 0.5 (clinical important difference) for subgroups (from stratum G) of new users with 2–3, 4–5, 6–8 or >8 HbA1c measures for both stable (< 2 HbA1c units) and unstable disease (≥ 2 HbA1c units), where prediction error is absolute difference between predicted and actual values of middle HbA1c observation and n is number of subjects being reported on, whose middle value was excluded.
**Figure S4c.** Proportion (%) with absolute prediction errors < 0.4 (measurement error) for subgroups (from stratum G) of new users with 2–3, 4–5, 6–8 or >8 HbA1c measures for both stable (< 2 HbA1c units) and unstable disease (≥ 2 HbA1c units), where prediction error is absolute difference between predicted and actual values of middle HbA1c observation and n is number of subjects being reported on, whose middle value was excluded.
**Figure S4d.** Mean squared absolute prediction errors for subgroups (from stratum G) of new users with 2–3, 4–5, 6–8 or >8 HbA1c measures for both stable (< 2 HbA1c units) and unstable disease (≥ 2 HbA1c units), where prediction error is absolute difference between predicted and actual values of middle HbA1c observation and n is number of subjects being reported on, whose middle value was excluded.
