# Supplementary material for: A comparison of methods for estimating the temporal change in a continuous variable: Example of HbA1c in patients with diabetes
Source: Pharmacoepidemiol Drug Saf. 2017 Aug 15;26(12):1474–82. doi: 10.1002/pds.4273 (PMC5724699; doi:10.1002/pds.4273)
Supplement: Supplementary file 1 — Table S1. Mean absolute prediction errors (MAPE), proportions of subjects with absolute prediction errors (APE) within clinical acceptability and measurement error, and mean squared absolute prediction errors (MSAPE), for each of eight strata. Reporting on subjects whose last HbA1c value excluded from analysis. Figure S1a. Mean absolute prediction errors for strata (A) new users on metformin only medication, (B) female new users, (C) female metformin‐only new users, (D) female metformin‐only new users aged a) ≤ 58, b) 59–70, c) ≥ 71 years, (E) new users with a) single medication change and b) two or more changes in medication, (F) new users whose distance in time between missing HbA1c measure and its closest neighbour was a) > 50th percentile b) > 75th percentile and c) > 90th percentile, where prediction error is absolute difference between predicted and actual values of last HbA1c observation and n is number of subjects being reported on, whose last value was excluded. Figure S1b. Proportion (%) with absolute prediction errors < 0.5 (clinical important difference) for strata (A) new users on metformin only medication, (B) female new users, (C) female metformin‐only new users, (D) female metformin‐only new users aged a) ≤ 58, b) 59–70, c) ≥ 71 years, (E) new users with a) single medication change and b) two or more changes in medication, (F) new users whose distance in time between missing HbA1c measure and its closest neighbour was a) > 50th percentile b) > 75th percentile and c) > 90th percentile, where prediction error is absolute difference between predicted and actual values of last HbA1c observation and n is number of subjects being reported on, whose last value was excluded. Figure S1c. Proportion (%) with absolute prediction errors < 0.4 (measurement error) for strata (A) new users on metformin only medication, (B) female new users, (C) female metformin‐only new users, (D) female metformin‐only new users aged a) ≤ 58, b) 59–70, c) ≥ 71 years, (E) new users [file PDS-26-1474-s001.docx]

**Supplementary Material**

**Table S1.** Mean absolute prediction errors (MAPE), proportions of subjects with absolute prediction errors (APE) within clinical acceptability and measurement error, and mean squared absolute prediction errors (MSAPE), for each of eight strata. Reporting on subjects whose last HbA1c value excluded from analysis.

|  | **LOCF** | **Global** | **Local** | **Bisector** | **AM** | **SLR** | **RE-no**  **covs** | **RE-with**  **covs***** | **FPCA** |
| --- | --- | --- | --- | --- | --- | --- | --- | --- | --- |
| *New users on metformin-only medication (n = 1,848)* | | | | | | | | | |
| **MAPE** | 0.43 | 1.05 | 1.07 | 1.03 | 0.46 | 0.69 | 0.45 | 0.45 | 0.42 |
| **APEs < 0.5*** | 71% | 49% | 49% | 47% | 69% | 61% | 71% | 71% | 74% |
| **APEs < 0.4**** | 63% | 42% | 42% | 40% | 61% | 52% | 63% | 63% | 67% |
| **MSAPE** | 0.52 | 4.48 | 6.01 | 4.36 | 0.54 | 2.42 | 0.52 | 0.52 | 0.49 |
| *Female metformin-only new users (n = 850)* | | | | | | | | | |
| **MAPE** | 0.42 | 1.07 | 1.12 | 1.07 | 0.45 | 0.72 | 0.44 | 0.44 | 0.41 |
| **APEs < 0.5*** | 73% | 50% | 50% | 48% | 71% | 62% | 73% | 73% | 76% |
| **APEs < 0.4**** | 65% | 43% | 43% | 41% | 64% | 53% | 65% | 65% | 68% |
| **MSAPE** | 0.54 | 5.68 | 8.76 | 6.17 | 0.57 | 3.92 | 0.55 | 0.54 | 0.51 |
| *New users with distance between missing & previous measures > 50^th^ percentile (n = 1,819)* | | | | | | | | | |
| **MAPE** | 0.65 | 1.93 | 2.06 | 1.95 | 0.70 | 1.28 | 0.68 | 0.68 | 0.64 |
| **APEs < 0.5*** | 58% | 35% | 34% | 33% | 54% | 43% | 57% | 57% | 60% |
| **APEs < 0.4**** | 50% | 29% | 29% | 28% | 48% | 37% | 50% | 50% | 53% |
| **MSAPE** | 1.14 | 14.41 | 27.13 | 16.06 | 1.17 | 8.27 | 1.14 | 1.13 | 1.07 |
| *New users with distance between missing & previous measures > 90^th^ percentile (n = 240)* | | | | | | | | | |
| **MAPE** | 0.87 | 3.19 | 3.44 | 3.29 | 0.84 | 2.62 | 0.84 | 0.83 | 0.80 |
| **APEs < 0.5*** | 50% | 26% | 27% | 27% | 50% | 27% | 51% | 52% | 55% |
| **APEs < 0.4**** | 43% | 22% | 23% | 23% | 43% | 23% | 42% | 42% | 48% |
| **MSAPE** | 2.05 | 39.60 | 54.07 | 42.46 | 1.76 | 30.02 | 1.65 | 1.62 | 1.69 |
| *New users with 2-3 measures + stable disease (n = 767)* | | | | | | | | | |
| **MAPE** | 0.45 | 1.19 | 1.19 | 1.19 | 0.44 | 0.91 | 0.43 | 0.43 | 0.43 |
| **APEs < 0.5*** | 64% | 40% | 40% | 40% | 64% | 47% | 66% | 66% | 65% |
| **APEs < 0.4**** | 56% | 34% | 34% | 34% | 56% | 39% | 59% | 59% | 58% |
| **MSAPE** | 0.37 | 3.77 | 3.77 | 3.77 | 0.35 | 2.22 | 0.33 | 0.33 | 0.33 |
| *New users with > 8 measures + stable disease (n = 51)* | | | | | | | | | |
| **MAPE** | 0.41 | 0.97 | 0.88 | 0.91 | 0.45 | 0.51 | 0.45 | 0.45 | 0.41 |
| **APEs < 0.5*** | 69% | 47% | 45% | 39% | 57% | 55% | 59% | 59% | 69% |
| **APEs < 0.4**** | 57% | 37% | 39% | 25% | 49% | 45% | 47% | 47% | 63% |
| **MSAPE** | 0.30 | 1.92 | 1.42 | 1.26 | 0.31 | 0.42 | 0.31 | 0.31 | 0.27 |
| *New users with 2-3 measures + unstable disease (n = 122)* | | | | | | | | | |
| **MAPE** | 1.89 | 6.07 | 6.07 | 6.07 | 2.01 | 4.60 | 1.80 | 1.77 | 1.79 |
| **APEs < 0.5*** | 20% | 5% | 5% | 5% | 11% | 9% | 13% | 19% | 9% |
| **APEs < 0.4**** | 17% | 4% | 4% | 4% | 8% | 7% | 11% | 12% | 6% |
| **MSAPE** | 5.35 | 96.95 | 96.95 | 96.95 | 5.17 | 76.53 | 4.53 | 4.46 | 4.18 |
| *New users with > 8 measures + unstable disease (n = 76)* | | | | | | | | | |
| **MAPE** | 0.86 | 2.79 | 2.35 | 2.48 | 1.17 | 1.47 | 1.17 | 1.17 | 0.96 |
| **APEs < 0.5*** | 46% | 25% | 22% | 21% | 25% | 18% | 25% | 24% | 39% |
| **APEs < 0.4**** | 42% | 21% | 18% | 17% | 22% | 17% | 20% | 20% | 34% |
| **MSAPE** | 1.70 | 16.67 | 12.64 | 10.33 | 2.27 | 3.49 | 2.19 | 2.19 | 1.78 |
| *clinically important differences; **measurement error; ***age & gender | | | | | | | | | |

**Figure S1a.** Mean absolute prediction errors for strata (A) new users on metformin only medication, (B) female new users, (C) female metformin-only new users, (D) female metformin-only new users aged a) ≤ 58, b) 59-70, c) ≥ 71 years, (E) new users with a) single medication change and b) two or more changes in medication, (F) new users whose distance in time between missing HbA1c measure and its closest neighbour was a) > 50^th^ percentile b) > 75^th^ percentile and c) > 90^th^ percentile, where prediction error is absolute difference between predicted and actual values of last HbA1c observation and n is number of subjects being reported on, whose last value was excluded.

LOCF (last occurrence carried forward), Global, Local and Bisector: Linear interpolation methods for value missing at the end.

AM: Arithmetic mean method.

SLR: Simple Linear Regression method.

RE-no covs: Random effects modelling method with no covariates included.

RE-with covs: Random effects modelling method with covariates included.

FPCA: Functional principal component analysis method.

**Figure S1b.** Proportion (%) with absolute prediction errors < 0.5 (clinical important difference) for strata (A) new users on metformin only medication, (B) female new users, (C) female metformin-only new users, (D) female metformin-only new users aged a) ≤ 58, b) 59-70, c) ≥ 71 years, (E) new users with a) single medication change and b) two or more changes in medication, (F) new users whose distance in time between missing HbA1c measure and its closest neighbour was a) > 50^th^ percentile b) > 75^th^ percentile and c) > 90^th^ percentile, where prediction error is absolute difference between predicted and actual values of last HbA1c observation and n is number of subjects being reported on, whose last value was excluded.

LOCF (last occurrence carried forward), Global, Local and Bisector: Linear interpolation methods for value missing at the end.

AM: Arithmetic mean method.

SLR: Simple Linear Regression method.

RE-no covs: Random effects modelling method with no covariates included.

RE-with covs: Random effects modelling method with covariates included.

FPCA: Functional principal component analysis method.

**Figure S1c.** Proportion (%) with absolute prediction errors < 0.4 (measurement error) for strata (A) new users on metformin only medication, (B) female new users, (C) female metformin-only new users, (D) female metformin-only new users aged a) ≤ 58, b) 59-70, c) ≥ 71 years, (E) new users with a) single medication change and b) two or more changes in medication, (F) new users whose distance in time between missing HbA1c measure and its closest neighbour was a) > 50^th^ percentile b) > 75^th^ percentile and c) > 90^th^ percentile, where prediction error is absolute difference between predicted and actual values of last HbA1c observation and n is number of subjects being reported on, whose last value was excluded.

LOCF (last occurrence carried forward), Global, Local and Bisector: Linear interpolation methods for value missing at the end.

AM: Arithmetic mean method.

SLR: Simple Linear Regression method.

RE-no covs: Random effects modelling method with no covariates included.

RE-with covs: Random effects modelling method with covariates included.

FPCA: Functional principal component analysis method.

**Figure S1d.** Mean squared absolute prediction errors for strata (A) new users on metformin only medication, (B) female new users, (C) female metformin-only new users, (D) female metformin-only new users aged a) ≤ 58, b) 59-70, c) ≥ 71 years, (E) new users with a) single medication change and b) two or more changes in medication, (F) new users whose distance in time between missing HbA1c measure and its closest neighbour was a) > 50^th^ percentile b) > 75^th^ percentile and c) > 90^th^ percentile, where prediction error is absolute difference between predicted and actual values of last HbA1c observation and n is number of subjects being reported on, whose last value was excluded.

LOCF (last occurrence carried forward), Global, Local and Bisector: Linear interpolation methods for value missing at the end.

AM: Arithmetic mean method.

SLR: Simple Linear Regression method.

RE-no covs: Random effects modelling method with no covariates included.

RE-with covs: Random effects modelling method with covariates included.

FPCA: Functional principal component analysis method.

**Figure S2a.** Mean absolute prediction errors for subgroups (from stratum G) of new users with 2-3, 4-5, 6-8 or >8 HbA1c measures for both stable (< 2 HbA1c units) and unstable disease (≥ 2 HbA1c units), where prediction error is absolute difference between predicted and actual values of last HbA1c observation and n is number of subjects being reported on, whose last value was excluded.

LOCF (last occurrence carried forward), Global, Local and Bisector: Linear interpolation methods for value missing at the end.

AM: Arithmetic mean method.

SLR: Simple Linear Regression method.

RE-no covs: Random effects modelling method with no covariates included.

RE-with covs: Random effects modelling method with covariates included.

FPCA: Functional principal component analysis method.

**Figure S2b.** Proportion (%) with absolute prediction errors < 0.5 (clinical important difference) for subgroups (from stratum G) of new users with 2-3, 4-5, 6-8 or >8 HbA1c measures for both stable (< 2 HbA1c units) and unstable disease (≥ 2 HbA1c units), where prediction error is absolute difference between predicted and actual values of last HbA1c observation and n is number of subjects being reported on, whose last value was excluded.

LOCF (last occurrence carried forward), Global, Local and Bisector: Linear interpolation methods for value missing at the end.

AM: Arithmetic mean method.

SLR: Simple Linear Regression method.

RE-no covs: Random effects modelling method with no covariates included.

RE-with covs: Random effects modelling method with covariates included.

FPCA: Functional principal component analysis method.

**Figure S2c.** Proportion (%) with absolute prediction errors < 0.4 (measurement error) for subgroups (from stratum G) of new users with 2-3, 4-5, 6-8 or >8 HbA1c measures for both stable (< 2 HbA1c units) and unstable disease (≥ 2 HbA1c units), where prediction error is absolute difference between predicted and actual values of last HbA1c observation and n is number of subjects being reported on, whose last value was excluded.

LOCF (last occurrence carried forward), Global, Local and Bisector: Linear interpolation methods for value missing at the end.

AM: Arithmetic mean method.

SLR: Simple Linear Regression method.

RE-no covs: Random effects modelling method with no covariates included.

RE-with covs: Random effects modelling method with covariates included.

FPCA: Functional principal component analysis method.

**Figure S2d.** Mean squared absolute prediction errors for subgroups (from stratum G) of new users with 2-3, 4-5, 6-8 or >8 HbA1c measures for both stable (< 2 HbA1c units) and unstable disease (≥ 2 HbA1c units), where prediction error is difference between absolute predicted and actual values of last HbA1c observation and n is number of subjects being reported on, whose last value was excluded.

LOCF (last occurrence carried forward), Global, Local and Bisector: Linear interpolation methods for value missing at the end.

AM: Arithmetic mean method.

SLR: Simple Linear Regression method.

RE-no covs: Random effects modelling method with no covariates included.

RE-with covs: Random effects modelling method with covariates included.

FPCA: Functional principal component analysis method.

**Table S2.** Mean absolute prediction errors (MAPE), proportions of subjects with absolute prediction errors (APE) within clinical acceptability and measurement error, and mean squared absolute prediction errors (MSAPE), for each of eight strata. Reporting on subjects whose middle HbA1c value excluded from analysis.

|  | **LOCF** | **FOCB** | **Global** | **Local** | **AM** | **SLR** | **RE-no**  **covs** | **RE-with**  **covs***** | **FPCA** |
| --- | --- | --- | --- | --- | --- | --- | --- | --- | --- |
| *New users on metformin-only medication (n = 1,848)* | | | | | | | | | |
| **MAPE** | 0.43 | 0.44 | 0.60 | 0.45 | 0.37 | 0.36 | 0.37 | 0.37 | 0.35 |
| **APEs < 0.5*** | 71% | 70% | 65% | 73% | 77% | 79% | 79% | 79% | 80% |
| **APEs < 0.4**** | 63% | 62% | 57% | 65% | 70% | 72% | 71% | 70% | 73% |
| **MSAPE** | 0.54 | 0.56 | 1.07 | 0.61 | 0.37 | 0.36 | 0.37 | 0.37 | 0.34 |
| *Female metformin-only new users (n = 850)* | | | | | | | | | |
| **MAPE** | 0.39 | 0.42 | 0.58 | 0.42 | 0.35 | 0.34 | 0.35 | 0.35 | 0.33 |
| **APEs < 0.5*** | 74% | 72% | 65% | 73% | 79% | 81% | 80% | 80% | 81% |
| **APEs < 0.4**** | 67% | 66% | 57% | 66% | 73% | 75% | 73% | 72% | 76% |
| **MSAPE** | 0.47 | 0.53 | 0.90 | 0.51 | 0.35 | 0.33 | 0.34 | 0.34 | 0.32 |
| *New users with distance between missing & previous measures > 50^th^ percentile (n = 1,819)* | | | | | | | | | |
| **MAPE** | 0.55 | 0.59 | 0.79 | 0.59 | 0.48 | 0.46 | 0.49 | 0.49 | 0.46 |
| **APEs < 0.5*** | 62% | 62% | 58% | 66% | 68% | 71% | 69% | 68% | 71% |
| **APEs < 0.4**** | 55% | 54% | 49% | 57% | 61% | 64% | 60% | 60% | 64% |
| **MSAPE** | 0.79 | 0.93 | 1.77 | 0.99 | 0.56 | 0.54 | 0.55 | 0.55 | 0.52 |
| *New users with distance between missing & previous measures > 90^th^ percentile (n = 240)* | | | | | | | | | |
| **MAPE** | 0.58 | 0.69 | 0.78 | 0.64 | 0.53 | 0.51 | 0.54 | 0.54 | 0.58 |
| **APEs < 0.5*** | 58% | 54% | 58% | 62% | 65% | 69% | 64% | 65% | 59% |
| **APEs < 0.4**** | 50% | 45% | 47% | 53% | 55% | 58% | 56% | 56% | 53% |
| **MSAPE** | 0.76 | 1.12 | 1.52 | 1.06 | 0.66 | 0.63 | 0.63 | 0.63 | 0.74 |
| *New users with 2-3 measures + stable disease (n = 767)* | | | | | | | | | |
| **MAPE** | 0.43 | 0.45 | 0.49 | 0.49 | 0.36 | 0.36 | 0.27 | 0.27 | 0.37 |
| **APEs < 0.5*** | 64% | 64% | 67% | 67% | 73% | 75% | 84% | 83% | 74% |
| **APEs < 0.4**** | 57% | 56% | 57% | 57% | 64% | 66% | 76% | 76% | 65% |
| **MSAPE** | 0.35 | 0.37 | 0.50 | 0.50 | 0.23 | 0.24 | 0.13 | 0.13 | 0.23 |
| *New users with > 8 measures + stable disease (n = 51)* | | | | | | | | | |
| **MAPE** | 0.34 | 0.44 | 0.71 | 0.48 | 0.35 | 0.33 | 0.40 | 0.40 | 0.32 |
| **APEs < 0.5*** | 76% | 65% | 43% | 71% | 75% | 82% | 67% | 67% | 75% |
| **APEs < 0.4**** | 67% | 61% | 35% | 65% | 55% | 63% | 55% | 55% | 73% |
| **MSAPE** | 0.19 | 0.36 | 0.88 | 0.43 | 0.20 | 0.17 | 0.25 | 0.25 | 0.19 |
| *New users with 2-3 measures + unstable disease (n = 122)* | | | | | | | | | |
| **MAPE** | 1.88 | 1.89 | 2.18 | 2.18 | 1.58 | 1.42 | 1.58 | 1.57 | 1.64 |
| **APEs < 0.5*** | 18% | 20% | 16% | 16% | 11% | 25% | 17% | 20% | 12% |
| **APEs < 0.4**** | 16% | 17% | 11% | 11% | 9% | 16% | 16% | 16% | 11% |
| **MSAPE** | 5.45 | 5.35 | 7.35 | 7.35 | 3.47 | 3.19 | 3.67 | 3.73 | 3.57 |
| *New users with > 8 measures + unstable disease (n = 76)* | | | | | | | | | |
| **MAPE** | 0.74 | 0.86 | 2.21 | 0.68 | 0.98 | 0.95 | 1.03 | 1.03 | 1.02 |
| **APEs < 0.5*** | 43% | 42% | 12% | 47% | 28% | 30% | 28% | 28% | 30% |
| **APEs < 0.4**** | 37% | 38% | 9% | 45% | 24% | 25% | 24% | 24% | 22% |
| **MSAPE** | 1.07 | 1.38 | 8.68 | 0.86 | 1.47 | 1.41 | 1.72 | 1.72 | 1.61 |
| *clinically important differences; **measurement error; ***age & gender | | | | | | | | | |

**Figure S3a.** Mean absolute prediction errors for strata (A) new users on metformin only medication, (B) female new users, (C) female metformin-only new users, (D) female metformin-only new users aged a) ≤ 58, b) 59-70, c) ≥ 71 years, (E) new users with a) single medication change and b) two or more changes in medication, (F) new users whose distance in time between missing HbA1c measure and its closest neighbour was a) >50^th^ percentile b) >75^th^ percentile and c) >90^th^ percentile, where prediction error is absolute difference between predicted and actual values of middle HbA1c observation and n is number of subjects being reported on, whose middle value was excluded.

LOCF (last occurrence carried forward), NOCB (next occurrence carried backward), Global and Local: Linear interpolation methods for value missing in the middle.

AM: Arithmetic mean method.

SLR: Simple Linear Regression method.

RE-no covs: Random effects modelling method with no covariates included.

RE-with covs: Random effects modelling method with covariates included.

FPCA: Functional principal component analysis method.

**Figure S3b.** Proportion (%) with absolute prediction errors < 0.5 (clinical important difference) for strata (A) new users on metformin only medication, (B) female new users, (C) female metformin-only new users, (D) female metformin-only new users aged a) ≤ 58, b) 59-70, c) ≥ 71 years, (E) new users with a) single medication change and b) two or more changes in medication, (F) new users whose distance in time between missing HbA1c measure and its closest neighbour was a) > 50^th^ percentile b) > 75^th^ percentile and c) > 90^th^ percentile, where prediction error is absolute difference between predicted and actual values of middle HbA1c observation and n is number of subjects being reported on, whose middle value was excluded.

LOCF (last occurrence carried forward), NOCB (next occurrence carried backward), Global and Local: Linear interpolation methods for value missing in the middle.

AM: Arithmetic mean method.

SLR: Simple Linear Regression method.

RE-no covs: Random effects modelling method with no covariates included.

RE-with covs: Random effects modelling method with covariates included.

FPCA: Functional principal component analysis method.

**Figure S3c.** Proportion (%) with absolute prediction errors < 0.4 (measurement error) for strata (A) new users on metformin only medication, (B) female new users, (C) female metformin-only new users, (D) female metformin-only new users aged a) ≤ 58, b) 59-70, c) ≥ 71 years, (E) new users with a) single medication change and b) two or more changes in medication, (F) new users whose distance in time between missing HbA1c measure and its closest neighbour was a) > 50^th^ percentile b) > 75^th^ percentile and c) > 90^th^ percentile, where prediction error is absolute difference between predicted and actual values of middle HbA1c observation and n is number of subjects being reported on, whose middle value was excluded.

LOCF (last occurrence carried forward), NOCB (next occurrence carried backward), Global and Local: Linear interpolation methods for value missing in the middle.

AM: Arithmetic mean method.

SLR: Simple Linear Regression method.

RE-no covs: Random effects modelling method with no covariates included.

RE-with covs: Random effects modelling method with covariates included.

FPCA: Functional principal component analysis method.

**Figure S3d.** Mean squared absolute prediction errors for strata (A) new users on metformin only medication, (B) female new users, (C) female metformin-only new users, (D) female metformin-only new users aged a) ≤ 58, b) 59-70, c) ≥ 71 years, (E) new users with a) single medication change and b) two or more changes in medication, (F) new users whose distance in time between missing HbA1c measure and its closest neighbour was a) > 50^th^ percentile b) > 75^th^ percentile and c) > 90^th^ percentile, where prediction error is absolute difference between predicted and actual values of middle HbA1c observation and n is number of subjects being reported on, with middle value excluded.

LOCF (last occurrence carried forward), NOCB (next occurrence carried backward), Global and Local: Linear interpolation methods for value missing in the middle.

AM: Arithmetic mean method.

SLR: Simple Linear Regression method.

RE-no covs: Random effects modelling method with no covariates included.

RE-with covs: Random effects modelling method with covariates included.

FPCA: Functional principal component analysis method.

**Figure S4a.** Mean absolute prediction errors for subgroups (from stratum G) of new users with 2-3, 4-5, 6-8 or >8 HbA1c measures for both stable (< 2 HbA1c units) and unstable disease (≥ 2 HbA1c units), where prediction error is absolute difference between predicted and actual values of middle HbA1c observation and n is number of subjects being reported on, whose middle value was excluded.

LOCF (last occurrence carried forward), NOCB (next occurrence carried backward), Global and Local: Linear interpolation methods for value missing in the middle.

AM: Arithmetic mean method.

SLR: Simple Linear Regression method.

RE-no covs: Random effects modelling method with no covariates included.

RE-with covs: Random effects modelling method with covariates included.

FPCA: Functional principal component analysis method.

**Figure S4b.** Proportion (%) with absolute prediction errors < 0.5 (clinical important difference) for subgroups (from stratum G) of new users with 2-3, 4-5, 6-8 or >8 HbA1c measures for both stable (< 2 HbA1c units) and unstable disease (≥ 2 HbA1c units), where prediction error is absolute difference between predicted and actual values of middle HbA1c observation and n is number of subjects being reported on, whose middle value was excluded.

LOCF (last occurrence carried forward), NOCB (next occurrence carried backward), Global and Local: Linear interpolation methods for value missing in the middle.

AM: Arithmetic mean method.

SLR: Simple Linear Regression method.

RE-no covs: Random effects modelling method with no covariates included.

RE-with covs: Random effects modelling method with covariates included.

FPCA: Functional principal component analysis method.

**Figure S4c.** Proportion (%) with absolute prediction errors < 0.4 (measurement error) for subgroups (from stratum G) of new users with 2-3, 4-5, 6-8 or >8 HbA1c measures for both stable (< 2 HbA1c units) and unstable disease (≥ 2 HbA1c units), where prediction error is absolute difference between predicted and actual values of middle HbA1c observation and n is number of subjects being reported on, whose middle value was excluded.

LOCF (last occurrence carried forward), NOCB (next occurrence carried backward), Global and Local: Linear interpolation methods for value missing in the middle.

AM: Arithmetic mean method.

SLR: Simple Linear Regression method.

RE-no covs: Random effects modelling method with no covariates included.

RE-with covs: Random effects modelling method with covariates included.

FPCA: Functional principal component analysis method.

**Figure S4d.** Mean squared absolute prediction errors for subgroups (from stratum G) of new users with 2-3, 4-5, 6-8 or >8 HbA1c measures for both stable (< 2 HbA1c units) and unstable disease (≥ 2 HbA1c units), where prediction error is absolute difference between predicted and actual values of middle HbA1c observation and n is number of subjects being reported on, whose middle value was excluded.

LOCF (last occurrence carried forward), NOCB (next occurrence carried backward), Global and Local: Linear interpolation methods for value missing in the middle.

AM: Arithmetic mean method.

SLR: Simple Linear Regression method.

RE-no covs: Random effects modelling method with no covariates included.

RE-with covs: Random effects modelling method with covariates included.

FPCA: Functional principal component analysis method.
